# Syntropic spin alignment at the interface between ferromagnetic and superconducting nitrides

**DOI:** 10.1093/nsr/nwae107

**Published:** 2024-03-19

**Authors:** Qiao Jin, Qinghua Zhang, He Bai, Meng Yang, Yonglong Ga, Shengru Chen, Haitao Hong, Ting Cui, Dongke Rong, Ting Lin, Jia-Ou Wang, Chen Ge, Can Wang, Yanwei Cao, Lin Gu, Guozhu Song, Shanmin Wang, Kun Jiang, Zhi-Gang Cheng, Tao Zhu, Hongxin Yang, Kui-juan Jin, Er-Jia Guo

**Affiliations:** Beijing National Laboratory for Condensed Matter Physics and Institute of Physics, Chinese Academy of Sciences, Beijing 100190, China; Beijing National Laboratory for Condensed Matter Physics and Institute of Physics, Chinese Academy of Sciences, Beijing 100190, China; Spallation Neutron Source Science Center, Dongguan 523803, China; Beijing National Laboratory for Condensed Matter Physics and Institute of Physics, Chinese Academy of Sciences, Beijing 100190, China; Department of Physics & Center of Materials Science and Optoelectronics Engineering, University of Chinese Academy of Sciences, Beijing 100049, China; Ningbo Institute of Materials Technology & Engineering, Chinese Academy of Sciences, Ningbo 315201, China; Beijing National Laboratory for Condensed Matter Physics and Institute of Physics, Chinese Academy of Sciences, Beijing 100190, China; Department of Physics & Center of Materials Science and Optoelectronics Engineering, University of Chinese Academy of Sciences, Beijing 100049, China; Beijing National Laboratory for Condensed Matter Physics and Institute of Physics, Chinese Academy of Sciences, Beijing 100190, China; Department of Physics & Center of Materials Science and Optoelectronics Engineering, University of Chinese Academy of Sciences, Beijing 100049, China; Beijing National Laboratory for Condensed Matter Physics and Institute of Physics, Chinese Academy of Sciences, Beijing 100190, China; Department of Physics & Center of Materials Science and Optoelectronics Engineering, University of Chinese Academy of Sciences, Beijing 100049, China; Beijing National Laboratory for Condensed Matter Physics and Institute of Physics, Chinese Academy of Sciences, Beijing 100190, China; Department of Physics & Center of Materials Science and Optoelectronics Engineering, University of Chinese Academy of Sciences, Beijing 100049, China; Beijing National Laboratory for Condensed Matter Physics and Institute of Physics, Chinese Academy of Sciences, Beijing 100190, China; Department of Physics & Center of Materials Science and Optoelectronics Engineering, University of Chinese Academy of Sciences, Beijing 100049, China; Institute of High Energy Physics, Chinese Academy of Sciences, Beijing 100049, China; Beijing National Laboratory for Condensed Matter Physics and Institute of Physics, Chinese Academy of Sciences, Beijing 100190, China; Department of Physics & Center of Materials Science and Optoelectronics Engineering, University of Chinese Academy of Sciences, Beijing 100049, China; Songshan Lake Materials Laboratory, Guangdong 523808, China; Beijing National Laboratory for Condensed Matter Physics and Institute of Physics, Chinese Academy of Sciences, Beijing 100190, China; Department of Physics & Center of Materials Science and Optoelectronics Engineering, University of Chinese Academy of Sciences, Beijing 100049, China; Songshan Lake Materials Laboratory, Guangdong 523808, China; Ningbo Institute of Materials Technology & Engineering, Chinese Academy of Sciences, Ningbo 315201, China; National Center for Electron Microscopy in Beijing and School of Materials Science and Engineering, Tsinghua University, Beijing 100084, China; Department of Physics, Southern University of Science and Technology, Shenzhen 518055, China; Department of Physics, Southern University of Science and Technology, Shenzhen 518055, China; Beijing National Laboratory for Condensed Matter Physics and Institute of Physics, Chinese Academy of Sciences, Beijing 100190, China; Department of Physics & Center of Materials Science and Optoelectronics Engineering, University of Chinese Academy of Sciences, Beijing 100049, China; Songshan Lake Materials Laboratory, Guangdong 523808, China; Beijing National Laboratory for Condensed Matter Physics and Institute of Physics, Chinese Academy of Sciences, Beijing 100190, China; Beijing National Laboratory for Condensed Matter Physics and Institute of Physics, Chinese Academy of Sciences, Beijing 100190, China; Department of Physics & Center of Materials Science and Optoelectronics Engineering, University of Chinese Academy of Sciences, Beijing 100049, China; Songshan Lake Materials Laboratory, Guangdong 523808, China; School of Physics, Zhejiang University, Hangzhou 310027, China; Beijing National Laboratory for Condensed Matter Physics and Institute of Physics, Chinese Academy of Sciences, Beijing 100190, China; Department of Physics & Center of Materials Science and Optoelectronics Engineering, University of Chinese Academy of Sciences, Beijing 100049, China; Songshan Lake Materials Laboratory, Guangdong 523808, China; Beijing National Laboratory for Condensed Matter Physics and Institute of Physics, Chinese Academy of Sciences, Beijing 100190, China; Department of Physics & Center of Materials Science and Optoelectronics Engineering, University of Chinese Academy of Sciences, Beijing 100049, China; Songshan Lake Materials Laboratory, Guangdong 523808, China

**Keywords:** magnetic proximity effects, nitride interfaces, polarized neutron reflectometry, superconductor/ferromagnetic interfaces, superconducting spintronics

## Abstract

The magnetic correlations at the superconductor/ferromagnet (S/F) interfaces play a crucial role in realizing dissipation-less spin-based logic and memory technologies, such as triplet-supercurrent spin-valves and ‘π’ Josephson junctions. Here we report the observation of an induced large magnetic moment at high-quality nitride S/F interfaces. Using polarized neutron reflectometry and DC SQUID measurements, we quantitatively determined the magnetization profile of the S/F bilayer and confirmed that the induced magnetic moment in the adjacent superconductor only exists below *T*_C_. Interestingly, the direction of the induced moment in the superconductors was unexpectedly parallel to that in the ferromagnet, which contrasts with earlier findings in S/F heterostructures based on metals or oxides. First-principles calculations verified that the unusual interfacial spin texture observed in our study was caused by the Heisenberg direct exchange coupling with constant J∼4.28 meV through *d*-orbital overlapping and severe charge transfer across the interfaces. Our work establishes an incisive experimental probe for understanding the magnetic proximity behavior at S/F interfaces and provides a prototype epitaxial ‘building block’ for superconducting spintronics.

## Teaser

The superconducting nitrides exhibited an unexpectedly parallel-aligned large magnetic moment across the interfaces when brought into proximity with a strong ferromagnetic nitride.

## INTRODUCTION

The interface between a superconductor (S) and a ferromagnet (F) is a topic of ongoing research in condensed matter physics. The interaction between superconductivity and ferromagnetism leads to fascinating phenomena, including (inverse) magnetic proximity effects [1–5], spin-triplet superconductivity [6,7], and the emergence of Majorana fermions [8]. Of particular interest is the magnetic proximity effect, which directly reflects the exchange interaction between the spins of electrons across S/F interfaces, resulting in the suppression of magnetic order or the appearance of unconventional superconductivity [9,10]. When a magnetic material is in proximity to a superconductor, the magnetic field can penetrate the superconductor over a short distance (usually a few nanometers) [11]. This causes spatial variation of the superconducting (SC) behavior near the interface and disrupts the Cooper pairs, significantly impacting the macroscopic physical properties of materials on both sides. Beyond the foundational comprehension established through S/F interfaces, there has been extensive exploration into the interplay between superconducting condensate states and magnetic-exchange spin coupling. This collective endeavor has given rise to an emerging realm of inquiry known as superconducting spintronics, which aims to develop dissipation-less spin-based devices, including memories [12], supercurrent spin-valves [13–15], and ‘π’ Josephson junctions [16]. Therefore, it is particularly relevant to experimentally verify the creation of a spin-polarized superconducting state with parallel or anti-parallel spin alignment at proximity-engineered S/F interfaces, as this is a crucial step in advancing this research direction.

So far, the underlying mechanism behind the magnetic proximity effect at S/F interfaces is still debated. Earlier research on S/F heterostructures composed of metal alloys reported an oscillation of the superconducting transition temperature as a function of the ferromagnetic layer thickness, demonstrating the possible unconventional propagation of superconducting pair waves in the system due to the strong exchange field [17–21]. With the development of advanced thin-film synthesis techniques, increasing attention has been focused on high-quality epitaxial S/F interfaces between a high-temperature superconductor (YBa_2_Cu_3_O_7_) and a fully spin-polarized half-metal ferromagnet (La_1−x_Ca_x_MnO_3_) [22–25]. Evidence from both X-ray magnetic circular dichroism (XMCD) [26] and polarized neutron reflectometry (PNR) [27–30] confirm a reduction of the magnetic moment at S/F heterointerfaces and the antiparallel spin alignment between Cu and Mn ions in each component. The magnetic proximity effects in YBa_2_Cu_3_O_7_/La_1−x_Ca_x_MnO_3_ interfaces are complex and depend on many impact factors, such as the electronic states of magnetic layers [26], thickness of the SC layer [29], nonhomogeneous domain structures [23,24], etc. Besides, the S/F heterointerfaces composed of superconducting VN and ferromagnetic alloy Pd_0.96_Fe_0.04_ had already been investigated [31–33]. The observed suppression of transition temperature, broadening of transition width, and spin-valve properties in S/F heterostructures suggest promising applications for superconducting spintronics. Although extensive studies have been conducted in both oxides, metals and nitrides, the opposite magnetic coupling through the S/F interfaces makes it difficult to control the spin collinearity by external magnetic fields. Furthermore, the experimental preliminary confirmation of the existence of the triplet component in S/F structures or the formation of a cryptoferromagnetic state in SC has seldom been explored. The active and reversible control of triplet supercurrents in spin-valve structures can be highly advantageous for developing superconducting spintronics in the future.

In this work, we investigated transition metal nitride bilayers composed of the superconducting VN and the ferromagnetic Fe_3_N using conventional electrical and magnetic transport measurements as well as PNR technique. Our findings indicate that the proximity of the SC layers to Fe_3_N can significantly reduce the upper critical fields and *T*_C_, resulting in unconventional magnetic behaviors. We also confirmed that an induced large magnetic moment extends a few nanometers into the SC layers, with its sign unexpectedly parallel to the magnetic moment in the Fe_3_N layer, which contradicts earlier reports and theoretical predictions. Instead of superexchange coupling at oxide interfaces, the Heisenberg direct exchange coupling at nitride interfaces is further verified by first-principles calculations. This study provides a detailed microscopic picture that offers intriguing insights into the magnetic correlations at S/F interfaces.

## RESULTS

### Fabrication of high-quality all-nitride heterostructures

**Figure 1.**
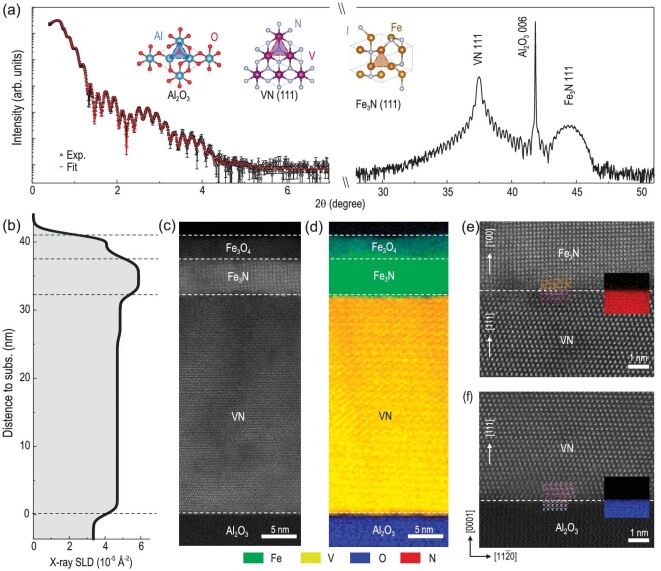
Atomically sharp interface between ferromagnetic Fe_3_N and superconducting VN. (a) XRR and *θ*-2*θ* scan of an Fe_3_N/VN bilayer grown on an Al_2_O_3_ substrate. The open symbols represent the experimental data, and the red line represents the best fit. The insets show the plane-view of crystal structures for all layers and substrates. (b) X-ray scattering length density (SLD) depth profile across the Fe_3_N/VN heterointerfaces. (c) High-angle annular dark-field scanning transmission electron microscopy (HAADF-STEM) image, and (d) combined electron-energy loss spectroscopy (EELS) spectrum image collected from a Fe_3_N/VN bilayer. The colors in the EELS mapping indicate the distribution of elements. Representative high-resolution TEM images at (e) Fe_3_N/VN and (f) VN/Al_2_O_3_ interfaces are also shown. The colored insets in (e) and (f) show the integrated intensities of N *K*- and O *K*-edges, respectively. The nitrogen content in Fe_3_N is comparably lower than that in VN, perhaps due to the unavoidable nitrogen vacancies during deposition. The atomic structures for each layer are shown for a better illustration.

The Fe_3_N/VN bilayer, as well as Fe_3_N and VN single films with different layer thicknesses, were grown on (00*l*)-oriented Al_2_O_3_ substrates by pulsed laser deposition (PLD) assisted by radio frequency (RF) nitrogen plasma (for experimental details, see Materials and Methods) [34–37]. Further details on the structural characterizations of the Fe_3_N and VN single layers can be found in our previous works [37,38]. Fig. 1(a) shows an XRD *θ*-2*θ* scan of the Fe_3_N/VN bilayer, which exhibits narrow diffraction peaks with high-order Laue thickness fringes, indicating the epitaxial growth of a high-quality bilayer. The full XRD *θ*-2*θ* scan of the bilayer is shown in Fig. S1, indicating both VN and Fe_3_N layers have a (111) orientation grown on Al_2_O_3_ substrates. There is no evidence of a pure Fe present in the films. The insets of Fig. 1(a) illustrate the in-plane atomic arrangements of the related nitrides and substrates. The epitaxial relationship between two nitride layers is enabled by their compatible crystallographic symmetry and relatively small misfit strain. X-ray reflectivity (XRR) measurements were used to examine the well-defined interfaces and each layer thickness. The root-mean-square (r. m. s.) roughness of the Fe_3_N/VN interface, averaged over the coherence of the X-ray beam projected on the sample's surface ($\approx $tens of millimeters^2^), was found to be 5.2 ± 0.5 Å. Fig. 1(b) shows the chemical depth profile obtained from fitting a model to XRR experimental data (with error bars). The X-ray scattering length density (SLD) profile revealed that the electron density at the surface of Fe_3_N is significantly lower compared to the interior part of the Fe_3_N layer. Additionally, the SLDs within the VN layer was nonuniform, with the SLD of the VN interfacial layer, having a thickness of 4.6 nm, greater by ∼5% compared to that of the rest of the VN layer. This effect should not be confused with a direct substitution of Fe into the VN structure (it is important to note that such chemical redistribution would not adequately explain the subsequent emergent state that will be discussed later).

We performed STEM measurements on the identical Fe_3_N/VN bilayer. Fig. 1(c) indicates the high crystallinity of both Fe_3_N and VN layers. The Fe_3_N surface layer has a lower HAADF brightness compared to the rest of the layer, consistent with XRR fitting results. Further investigation of the chemical composition was carried out using element-specific electron energy loss spectroscopy (EELS) and energy dispersive X-ray spectroscopy (EDX), as shown in Fig. 1(d) and Figs. S2–S4. We discovered that the Fe_3_N surface layer contains a significant amount of oxygen, indicating the oxidization of Fe ions. A high-magnification STEM image was taken at a representative surface region of the Fe_3_N/VN bilayer (Fig. S5), revealing that the top surface has a spinel (Fe_3_O_4_) structure with clearly visible octahedral and tetrahedral iron atomic columns. Therefore, the top layer of the Fe_3_N/VN bilayer comprises a mixture of Fe_3_N and Fe_3_O_4_ films. The oxidization of the Fe_3_N layer is an unavoidable phenomenon when exposed to ambient conditions, which is commonly observed in other Fe nitride compounds [39]. Notably, the Fe_3_O_4_ surface layer is not fully crystallized, rather that some parts of surface are amorphous or polycrystalline. The surface Fe_3_O_4_ layer does not contribute to the XRD signals. We want to mention that the Fe_3_O_4_ surface layer does not affect the quality of the buried interfaces. The atomic-resolved interfaces of Fe_3_N/VN and VN/Al_2_O_3_ are shown in Figs. 1(e) and 1(f), respectively. Both interfaces exhibit well-aligned atoms (insets, crystal structures are illustrated) and negligible chemical intermixing inside the VN layers.

Additionally, XAS measurements were performed at room temperature with a small incident angle (substantial portion of irradiated area). The spectra from multiple samples were collected at the N *K*-, V *L*-, and Fe *L-*edges for comparison (Fig. S6). The XAS intensity at the N *K*-edge from Fe_3_N/VN is evident but noticeably lower compared to VN alone. Control measurements confirm that the predominant N signal originates from the Fe_3_N layer rather than the underlying VN layer, aligning with our earlier XPS findings on a Fe_3_N single layer [38]. Furthermore, we have observed a shift in the valence state of V ions away from +3 towards a lower valence state, indicating electron doping across the interfaces. Simultaneously, the valence state of Fe ions also undergoes changes, with the peak positions moving to a lower energy. Notably, a significant disparity in the line shapes of XAS at Fe *L*-edges between Fe and Fe_3_N suggests that the Fe_3_N layer is not composed of pure Fe. This finding bears resemblance to observations made at the YBa_2_Cu_3_O_7_/La_1-x_Ca_x_MnO_3_ interfaces, where XAS measurements confirmed significant charge transfer between cations across the interfaces [24].

### Suppression of superconductivity in proximity to Fe_3_N

**Figure 2.**
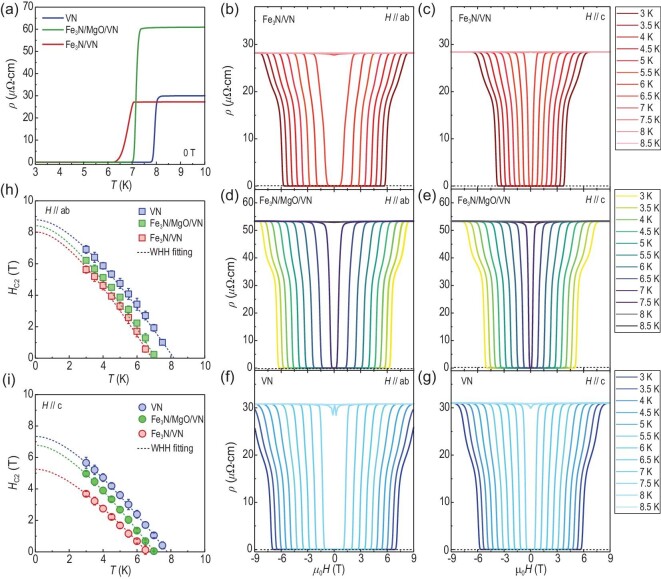
Suppression of superconducting transition temperature (*T*_C_) by a strong ferromagnet Fe_3_N. (a) Temperature-dependent resistivity of a VN single layer and a Fe_3_N/VN bilayer at zero magnetic field. The *T*_C_ of the VN films was suppressed by ∼1.55 K. (b) and (c) Magnetic-field dependent resistivity of the Fe_3_N/VN bilayer at various temperatures when the magnetic field was applied parallel to the *ab*-plane and the *c*-axis, respectively. Similar measurements were performed on the Fe_3_N/MgO/VN trilayer and the VN single layer, as shown in (d) and (e), and (f) and (g), respectively. (h, i) Temperature dependence of the upper critical field (*H*_C2_) for the VN single layer, Fe_3_N/VN bilayer and Fe_3_N/MgO/VN trilayer. The square and circle symbols represent *H*_C2_ when the magnetic field was applied parallel to (h) the *ab*-plane and (i) the *c*-axis. The dashed lines are fitting curves based on the Werthamer-Helfand-Hohenberg (WHH) model.

Fig. 2(a) presents the temperature-dependent resistivity of a VN single film and a Fe_3_N/VN bilayer at zero magnetic field. Prior to the sharp superconducting transition, all samples exhibit metallic phases. The normal state Hall resistivity measured at 10 K confirms that the charge carriers are electrons (Fig. S7) and reveals that the carrier densities of the VN single film and Fe_3_N/VN bilayer fall within the range of (19.1–26.3) × 10^22^ cm^−3^ (Table 1). The superconducting transition temperature (*T*_C_) of the VN single film is ∼7.78 K and is suppressed by ∼1.55 K after capping a ferromagnetic Fe_3_N layer. This effect is typically attributed to the presence of spin-polarized quasiparticles at the interface, which can enhance the scattering of the Cooper pairs responsible for superconductivity [31–33]. Temperature-dependent resistivity of the two samples were recorded under different magnetic fields, both parallel and perpendicular to the sample's surface plane, up to 9 T (Fig. S8). Both samples exhibit conventional suppressed superconductivity with increasing field. The VN is a typical type-II superconductor with upper critical field (*H*_c2_) [40]. Figs. 2(b) and 2(c) show the in-plane and out-of-plane magnetic field-dependent resistivity of a Fe_3_N/VN bilayer, with temperature fixed at values from 3 K to 8.5 K, respectively. The transport measurements were conducted repeatedly on a Fe_3_N/MgO/VN trilayer [Figs. 2(d) and 2(e)] and a VN single film [Figs. 2(f) and 2(g)]. The *H*_c2_ is determined at fields when resistivity decreases to the half value of its normal state. In Figs. 2(h) and 2(i), we plotted the temperature dependence of the *H*_c2_ for three samples when *H*//*ab* and *H*//*c*, respectively. Notably, all samples exhibit a smaller *H*_c2_ (*H*//*c*) compared to *H*_c2_ (*H*//*ab*). These data can be well-fitted by a single-band Werthamer-Herlfand-Hohenberg (WHH) equation (dashed lines) [41]. The fitting parameters are summarized in Table 1. The mean free path (λ) of the VN layer increases by ∼30% after proximity to a Fe_3_N layer, whereas the estimated coherence length (***ξ***_GL_) increases from ∼6.7 nm (VN) to ∼7.9 nm (Fe_3_N/VN) obtained from Ginzburg-Landau (GL) formula, ${\mu }_0{H}_{C2}( 0 ) = \frac{{{\phi }_0}}{{2\pi \xi _{GL}^2}}$, where ${\phi }_0$=2.068 × 10^−15^ Wb. An additional crucial observation is that the MgO spacer layer effectively hinders the strong interaction between Fe_3_N and VN. We posit that the impact of ferromagnetic Fe_3_N on the superconducting state in VN diminishes with the progressive increase in the thickness of the MgO layer.

**Table 1.** Summary of the parameters for the VN superconducting single layers, Fe_3_N/VN bilayers and Fe_3_N/MgO/VN trilayers, including layer thickness (*t*), superconducting transition temperature (*T*_C_), upper critical field (*H*_C2_), carrier density (*n*), Fermi vector (*κ_F_*), coherence length (***ξ***_GL_), and mean free path (λ). The *L*_C_ is obtained from Ginzburg-Landau (GL) coherence length formula, ${\mu }_0{H}_{C2}( 0 ) = \frac{{{\phi }_0}}{{2\pi \xi _{GL}^2}}$, where ${\phi }_{0{\mathrm{\ }}}$ = 2.068×10^−15^ Wb. The electronic parameters, such as resistivities, carrier densities, and mobilities, of single layer and bilayers were extracted from transport measurements conducted in the normal state at 10 K (Fig. S7). Estimated errors are listed in parentheses.

	VN	VN	Fe_3_N/MgO/VN	Fe_3_N/VN
*t* (nm)	6.0(1)	33(1)	6.5(1)/5(1)/33(1)	6.5(1)/33(1)
*T*_C_ (K)	4.5(5)	7.78(3)	7.12(2)	6.23(2)
*H*_C2_ (*H*//*c*) (T)	2.95(6)	7.34(5)	6.89(2)	5.25(5)
*H*_C2_ (*H*//*ab*) (T)	11.47(5)	8.74(3)	8.42(1)	8.01(5)
*n* (×10^22^ cm^−3^)	17.5(3)	26.3(2)	24.2(1)	19.1(3)
*κ_F_* (10^6^ m/s)	2.0(4)	2.29(2)	2.16(1)	2.09(2)
*ξ*_GL_ (nm)	10.6(0.1)	6.7(0.1)	6.9(0.1)	7.9(0.1)
*λ* (nm)	0.86(2)	1.03(5)	1.12(1)	1.32(4)

### Induced magnetization in the interfacial VN layers

**Figure 3.**
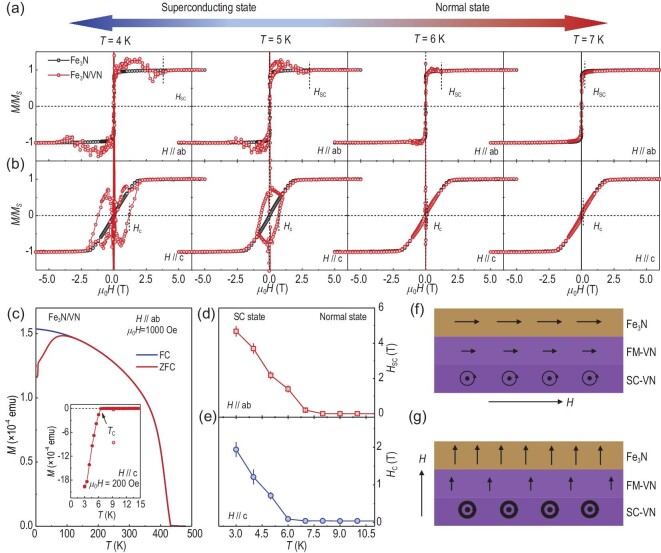
Strong magnetic coupling at the interfaces between Fe_3_N and VN. (a) and (b) *M-H* hysteresis loops measured from the Fe_3_N single layer and Fe_3_N/VN bilayer, respectively, with magnetic fields applied parallel to the *ab*-plane and *c*-axis. The temperature for *M-H* loops was varied across the superconducting transition. The magnetizations were normalized to *M*_S_. (c) *M-T* curves of a Fe_3_N/VN bilayer. The measurements were carried out during the sample warm-up after zero-field cooling (ZFC) and field cooling (FC) at 1 kOe. The inset of (c) shows the *M-T* curve of a Fe_3_N/VN bilayer when an out-of-plane magnetic field of 200 Oe was applied. Temperature dependence of (d) the critical field (*H*_SC_) and (e) coercive field (*H*_C_) under in-plane and out-of-plane magnetic fields. (f) and (g) Illustrations of the spin alignment and magnetic vortices within Fe_3_N and VN layers when magnetic fields are parallel or perpendicular to the interfaces, respectively. The interface region of VN presents a ferromagnetic state. The illustrations of the vortices provide a guide for the eyes. In reality, the orientations of vortices should be perpendicular to the applied fields.

Earlier studies have shown that the magnetic state of the superconducting VN layer can be modified due to the proximity effect [1–5]. To test this hypothesis, we measured the field- and temperature-dependent magnetization (*M*) of the Fe_3_N and VN single layer, and a Fe_3_N/VN bilayer [Figs. 3(a), 3(b), 3(c), and Fig. S9]. Please note that the surface Fe_3_O_4_ layer exhibiting a lower magnetization may slightly influence the saturation magnetization [42–44], but not affecting the exchange interaction between Fe_3_N and VN. The *M-T* curve of Fe_3_N/VN bilayer confirms the presence of diamagnetic signals when the VN layer enters the SC state [Fig. 3(c)]. The *T*_C_ obtained from the *M-T* curves is consistent with the electrical transport measurements [inset of Fig. 3(c)]. We recorded both in-plane and out-of-plane *M* vs *H* at fixed temperatures across *T*_C_. The magnetic easy-axis is along the in-plane direction [38]. At the high field regions, *M* of both samples is saturated. Although we observed that the saturation moment (*M*_sat_) of a Fe_3_N/VN bilayer differs from that of a Fe_3_N single layer, there could be multiple reasons contributing to this discrepancy, such as variations in the thickness of the oxidized surface layer and the quality of the Fe_3_N layer. To enable a meaningful comparison of the *M-H* curves between the Fe_3_N single layer and the Fe_3_N/VN bilayer, we normalize *M* of both samples to *M*_sat_. At the low field region, we observe abnormal behavior in the hysteresis loops of the Fe_3_N/VN bilayer when the temperature is lowered below *T*_C_. When *H*//*ab*, the magnetic moment initially increases with applied fields and then returns to a constant value of *M*_sat_ beyond a critical field (*H*_SC_). This behavior suggests the presence of an induced magnetic moment in the VN layer for temperatures below *T*_C_. Simultaneously, the out-of-plane magnetic moment of the Fe_3_N/VN bilayer jumps to incredible values at small fields and exhibits an increased coercive field (*H*_C_) when the VN layer is in the superconducting state. Figs. 3(d) and 3(e) show *H*_SC_ and *H*_C_ as a function of the temperature, respectively. Clearly, *H*_SC_ and *H*_C_ in the SC state reduce progressively as the temperature increases. When the temperature is above *T*_C_, the anomaly in the magnetization along both the in-plane and out-of-plane directions disappears, aligning with the magnetic behavior of a Fe_3_N single layer. As an additional experiment, we investigated the Ta/MgO/Fe/V multilayer, which did not exhibit superconducting behavior, and the magnetization was significantly higher than that of a Fe_3_N/VN bilayer (Fig. S10).

**Figure 4.**
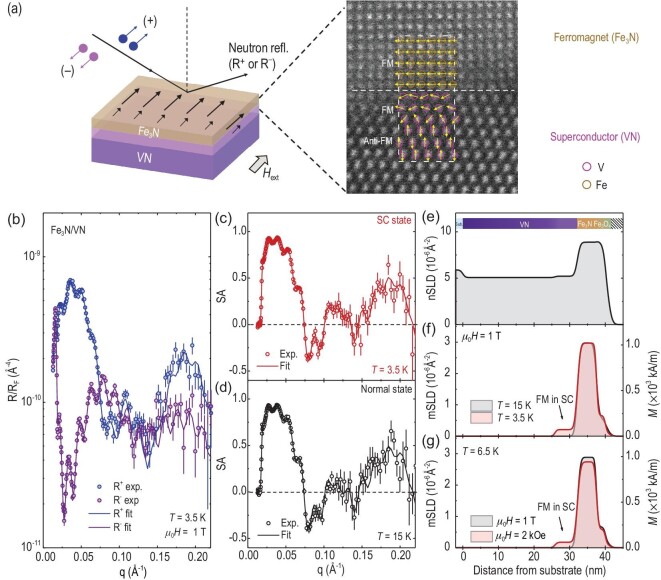
Magnetic depth profiling across Fe_3_N/VN heterostructures. (a) Schematic of PNR set-up. PNR measurements were performed at fixed temperatures under external in-plane magnetic fields. The inset shows a high-resolution HAADF-STEM image at the interface region of a Fe_3_N/VN bilayer, with illustrated spin textures. (b) Measured (open symbols) and fitted (solid lines) reflectivity curves for spin up (*R*^+^) and spin down (*R*^–^) polarized neutrons as a function of wave vector (*q*). The reflectivities were normalized to the Fresnel reflectivity (*R*_F_). PNR spin-asymmetry (SA) ratio SA = (*R*^+^+ *R*^–^)/(*R*^+^−*R*^–^) obtained from the experimental and fitted reflectivities when VN is in (c) the superconducting (SC) state (*T* = 3.5 K) and (d) the normal state (*T* = 15 K) under an external magnetic field of 1 T. The error bars represent one standard deviation. Fits in (c) with zero and negative magnetization in the VN interfacial layer are shown with dashed lines, demonstrating large deviations from experimental data. (e) Nuclear scattering length density (nSLD) and (f) magnetic scattering length density (mSLD) profiles measured for a Fe_3_N/VN bilayer at 3.5 and 15 K were presented as a function of the distance from substrate. The scale on the right-hand side shows the absolute magnetization (*M*). (g) PNR mSLD profiles of a Fe_3_N/VN bilayer at *T* = 6.5 K with magnetic fields of 2 kOe and 1 T. The magnetization measured inside the VN layer in (f) and (g) is marked with arrows.

To quantitatively determine the interfacial magnetization profile, we performed systematic PNR measurements on a Fe_3_N/VN bilayer, as shown in Fig. 4(a). The specular reflectivity (*R*) of the bilayer was measured as a function of the wave vector transfer (*q*), with *R*^+^ and *R*^–^ representing the reflectivities for neutrons with spins parallel or anti-parallel to the applied magnetic field, respectively. The PNR measurements were performed under a magnetic field of 1 T at 3.5 K, when the Fe_3_N/VN bilayer was in the SC state. Fig. 4(b) shows the PNR data with the reflectivity normalized to the asymptotic value of the Fresnel reflectivity *R*_F_ (=16π^2^/q^4^). The large *q*-dependent splitting between the two reflectivities reflects the large net magnetization of the Fe_3_N/VN bilayer. To fit the PNR data, we used the chemical depth profile obtained from XRR fitting to strictly constrain a model for PNR fitting. We obtained the nuclear and magnetic SLD profiles corresponding to the chemical and magnetization distribution as a function of film thickness, as shown in Figs. 4(e) and 4(f). The best fits demonstrate that the Fe_3_N layer has a magnetization of 1019.3 ± 5.4 kA/m and that the Fe_3_O_4_ surface layer has a much lower magnetization of 212.2 ± 5.1 kA/m. The magnetization of Fe_3_N interior layer is slightly larger than the magnetization measured by SQUID (∼915 kA/m), which underestimated the value by assuming a uniform distribution of magnetization across the entire Fe_3_N layer. Additionally, we observed that the VN interfacial layer in close proximity to Fe_3_N exhibits a significant net moment of 72.3 ± 2.2 kA/m, which aligns parallel to the Fe_3_N magnetization. The spin texture schematic at the Fe_3_N/VN interfaces is depicted in Fig. 4(a). Confidence in this interpretation of syntropic spin alignment at the interface is further reinforced by fitting the spin asymmetry (SA) curve derived from experimental data [Fig. 4(c)]. To fit the PNR-SA data, we considered three different scenarios: (1) parallel spins (positive *M*); (2) no spins (zero *M*); and (3) antiparallel spins (negative *M*) of the VN interfacial layer with respect to the spins of Fe_3_N layer (Fig. S11). The optimal fit to the PNR data, characterized by the lowest χ^2^ metric of ∼1.1, followed by χ^2^ metrics of ∼1.6 (zero *M*) and ∼1.8 (negative *M*), serves as compelling evidence, underscoring the inherent ferromagnetic coupling across the interface. The 95% confidence intervals for the fitting magnetizations are summarized in the parentheses of Table 2. Under the same magnetic field of 1 T, we conducted PNR measurements on the Fe_3_N/VN bilayer at 15 K [Fig. 4(d)], where the VN enters normal state. The induced magnetic moment in the VN interfacial layer decreases to zero [Fig. 4(f)], which is consistent with SQUID results.

**Table 2.** Summary of the XRR and PNR results for a Fe_3_N/VN bilayer. Median parameter values and 95% confidence limits were determined by a Markov Chain Monte Carlo (MCMC) fit of the simulated data.

*T*	*μ*_0_H	*t*_Fe_3_O_4__	*t*_Fe_3_N_	*t*_VN interface_	*t*_VN bulk_	*M*_Fe_3_O_4__	*M*_Fe_3_N_	*M*_VN interface_	*M*_VN bulk_
(K)	(T)	(nm)	(nm)	(nm)	(nm)	(kA/m)	(kA/m)	(kA/m)	(kA/m)
3.5	1	1.7 (0.3)	4.8 (0.6)	4.6 (0.6)	27.7 (1.0)	212.2 (5.1)	1019.3 (5.4)	72.3 (2.2)	0
15	1					177.5 (7.9)	1006.7 (3.7)	3.5 (2.5)	0
6.5	0.2					134.7 (5.9)	939.7 (6.3)	60.3 (2.4)	0
6.5	1					191.6 (6.2)	992.3 (7.8)	2.4 (2.2)	0

To confirm the observation of the induced moment in the superconducting VN interfacial layer, we performed further control measurements on the Fe_3_N/VN bilayer at a fixed temperature of 6.5 K with varying magnetic fields between 2 kOe and 1 T (Fig. S12). The measuring temperature was carefully chosen at the boundary of the phase transition between the SC state and normal state. Under a magnetic field of 2 kOe, the Fe_3_N/VN bilayer remained in the SC state. The VN interfacial layer exhibits a net magnetic moment of 60.3 ± 2.4 kA/m [Fig. 4(g)]. As the magnetic field was increased to 1 T, the magnetization of the Fe_3_N layer increased only by ∼5% because the Fe_3_N is nearly saturated due to the small *H*_C_. However, no induced moment in the VN interfacial layer was observed. This was because the SC state of the bilayer was destroyed, and it enters the normal state at high magnetic fields. Therefore, our experimental results provide unambiguous evidence that a parallel magnetic moment in the VN layer close to the interface only exists once the bilayer enters the SC state. At this moment, we could not argue the formation of magnetic domains below *T*_C_ due to the presence of magnetic vortices creating electrodynamic forces [23,45,46]. The off-specular PNR experiments did not show the clean Bragg peak diffraction below *T*_C_. At least, our results clearly rule out the existence of a triplet supercurrent at the interface that previously had been proposed to explain the observed magnetism at S/F interfaces [47–49], otherwise the induced moment of VN interfacial layer should have an opposite sign with respect to that of Fe_3_N.

**Figure 5.**
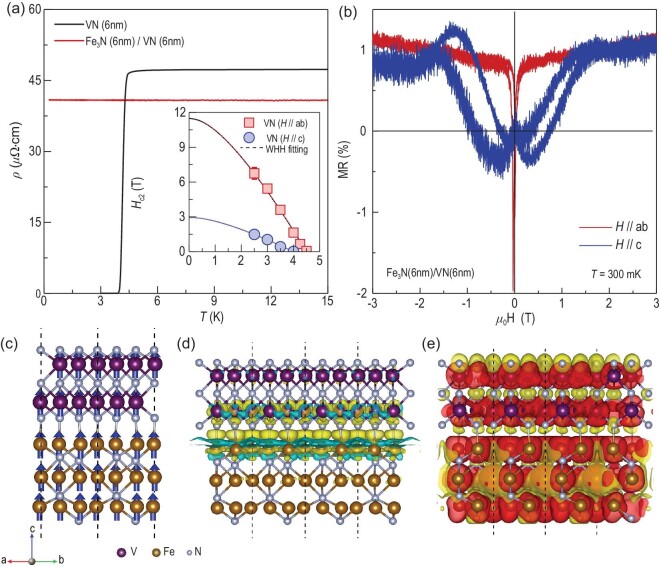
First-principles calculations on the electronic and spin states across Fe_3_N/VN interfaces. (a) Temperature-dependent resistivity of a 6-nm-thick VN single layer and a Fe_3_N/VN (6 nm) bilayer at zero magnetic field. The bilayer maintains metallic phase down to 300 mK, suggesting the ferromagnetic Fe_3_N completely suppressed the superconductivity. Inset shows the temperature dependence of *H*_C2_ for the 6-nm-thick VN single layer. The square and circle symbols represent *H*_C2_ when the magnetic field was applied parallel to the *ab*-plane and the *c*-axis, respectively. The dashed lines are fitting curves based on the WHH model. (b) Field-dependent magnetoresistance (MR) of a Fe_3_N/VN (6 nm) bilayer at 300 mK. Both in-plane and out-of-plane MR are recorded. (c) Side-view of the interface region in a Fe_3_N/VN bilayer. The blue arrows indicate the spin orientation of ions. (d) Charge density differential distributions for FM state at the interface when the charge density is equal to ±5 × 10^−3^ e/Å^3^. The colored illustrations indicate the isosurfaces, corresponding to the charge accumulation (yellow) and depletion (green) in the space. (e) Contour density corresponding to the FM state for spin up (red) and spin down (yellow) states when the spin density is equal to ± 3 × 10^−3^ μ_B_/Å^3^.

Furthermore, earlier work demonstrates that the magnetic exchange coupling in the YBa_2_Cu_3_O_7_/La_1-x_Ca_x_MnO_3_ heterostructures strongly depends on the thickness of superconducting layer [23,26,50]. We measured the transport properties of a VN single film and a Fe_3_N/VN bilayer with a VN layer thickness of ∼6 nm [Fig. 5(a)]. The 6-nm-thick VN film exhibits a superconducting behavior with *T*_C_ ∼4.5 K and a severely increased in-plane anisotropy due to geometrical constraints (Fig. S13). Moreover, the Fe_3_N/VN bilayer maintains the ferromagnetic metallic phase with clear anisotropic MR when the temperature is lowered down to 300 mK [Figs. 5(b) and S14]. These results suggest the strong suppression of superconducting ordering by a ferromagnetic capping layer. We note that the induced ferromagnetism and superconductivity cannot coexist macroscopically in the interfacial VN layers.

### First-principles calculations on interfacial proximity effects

Previous experimental evidence of long-range penetrated ferromagnetism into conventional superconducting oxides reveals an antiparallel magnetic coupling with respect to the ferromagnetic layers [23–30]. In those cases, the superexchange coupling between 3*d* transitional metal elements via oxygen 2*p* orbitals results in an antiferromagnetic spin alignment. However, our findings demonstrate that spins can be aligned parallel to each other across S/F interfaces [Fig. 5(c)]. We performed the first-principles calculations on Fe_3_N/VN interfaces based on density functional theory (DFT). Both ferromagnetic (FM) and antiferromagnetic (AFM) configurations are initially textured (Fig. S15). We calculated the Heisenberg exchange coupling constant (*J*) between Fe and V atoms near the interface to be 4.28 meV. The positive sign indicates the ferromagnetic coupling across the interfaces. Besides, we find that the electron clouds (marked in yellow) around the interfaces is denser than those in the interior parts of VN and Fe_3_N layers [Fig. 5(d)]. The calculation results indicate the electron transfer from Fe to V ions across the interface due to the charge density difference. This dynamic charge redistribution, driven by interfacial wavefunction overlap, can significantly influence exchange interactions and spin-orbit coupling, thereby modulating the magnetic characteristics of VN and culminating in a congruous spin alignment. The calculation results are in a good agreement with our XAS results (Fig. S6). We further calculated the contour density for the spin up (red) and spin down (yellow) components in Fig. 5(e). We found that the spin polarization exhibits a large positive value for the constructed heterointerface. The density of states (DOS) for spin-up channels are significantly larger than those of spin-down channels at the Fermi level, consistently implying that the heterointerfaces exhibit ferromagnetic coupling.

The ferromagnetic spin alignment between Fe and V ions is in sharp contrast to earlier works on the spin coupling at oxide interfaces. We propose a qualitative physical explanation for the observed magnetic behaviors at the Fe_3_N/VN interfaces. When the temperature falls below *T*_C_, magnetic vortices form in the superconducting VN layer, but they do not influence the in-plane magnetization of the bilayer [Fig. 3(f)]. In the Fe_3_N/VN heterostructures with strong exchange fields, the number of spin-up and spin-down electrons is imbalanced, leading to a magnetic ‘leakage’ from the Fe_3_N layer to into the VN layer and electron polarization in the VN layer persists over a short length scale. In this scenario, the direction of the induced magnetic moment in the VN interfacial layer aligns parallel to that in the Fe_3_N layer. This finding is supported by numerical solutions to the Bogoliubov–de Gennes equations [2,3], which are consistent with our SQUID and PNR results. However, when the magnetic field switches to the out-of-plane direction [Fig. 3(g)], a different situation arises. At small fields, the Fe_3_N/VN bilayer remains in SC state, and the magnetic flux will be blocked or penetrate through the vortices. This leads to the observation of incredibly large magnetic moments in the Fe_3_N/VN bilayer at small magnetic fields. When the magnetic field exceeds *H*_c2_ or the temperature increases above *T*_C_, the Fe_3_N/VN bilayer enters the normal state, the impact from the superconducting VN layer vanishes, thus greatly weakening the strength of the interfacial magnetic coupling. The *M*_sat_ and *H*_C_ of the Fe_3_N/VN bilayer return to the values that are nearly identical to those of a Fe_3_N single layer.

## DISCUSSION AND CONCLUSION

In summary, our work presents new findings regarding the induced magnetic moment in a superconducting VN layer in proximity to a strong ferromagnetic Fe_3_N layer. Our results differ from previous studies, as we have discovered that the spin orientation in VN is ferromagnetically coupled to the magnetization of the adjacent Fe_3_N layer, revealing a unique form of magnetic coupling across the S/F interface. Our research sheds light on the potential for exploring complex competing orders in the large family of transition metal nitrides, such as ZrN and NbN [51,52], which are known to host superconducting transitions. Such materials have not been thoroughly investigated in the past, and our study provides a strategy for future exploration in superconducting spintronics. Additionally, we recommend prioritizing the testing of binary or perovskite-type nitrides containing 5*d* elements [53,54]. These materials not only have competing phases, but also exhibit a strong spin-orbital (SO) interaction. This interaction has an energy scale comparable to the superconducting gap, which may have a significant impact on the penetration length of the triplet component into the superconductor, thereby dominating the long-range proximity effect in S/F structures.

## METHODS

### Sample synthesis

The VN and Fe_3_N thin films were fabricated on (001)-oriented single-crystalline Al_2_O_3_ substrates by PLD. Stoichiometric binary nitride targets were synthesized using a high-pressure reaction route at the high-pressure Laboratory of Southern University of Science and Technology (SUSTech). The nitride single films had been fabricated and characterized (structure, chemical, and functionality) independently in our previous works [37,38]. The VN films were deposited at a substrate temperature of 750°C, while the Fe_3_N films were deposited at a lower substrate temperature of 300°C to maintain their high crystallinity. During deposition, the density of laser fluence was maintained at ∼ 1 J/cm^2^, and the base pressure was kept at around 10^−8^ Torr. An RF plasma source with tunable input power (100–400 W) and partial pressure of N_2_ gas (10^−3^–10^−6^ Torr) was applied to generate highly active nitrogen atoms. The generated nitrogen plasma helped to compensate the nitrogen vacancies in the films, enabling the as-grown films to largely maintain their intrinsic characteristics. To cross-check the interface properties, we inserted an ultrathin MgO layer between the Fe_3_N and VN using PLD, forming a Fe_3_N/MgO/VN trilayer. The growth conditions for MgO layers are identical to that of the VN layer. The thickness of the MgO layer is approximately 3 nm. Additionally, we fabricated a Ta/MgO/Fe/V multilayer on Al_2_O_3_ substrates using magnetron sputtering. This sample is used as a control measure to investigate the different physical properties between pure metals and nitrides. The thicknesses of nitrides (VN, Fe_3_N), metals (V, Fe), and MgO layers were controlled by counting laser pulses and sputtering time.

### Structural and electronic characterizations

XRR measurements were conducted to confirm the structural integrity, layer thickness, interface/surface roughness, and densities of all layers, from which we could obtain the chemical profiles of Fe_3_N/VN bilayers. The *θ*-2*θ* scans were performed on a high-resolution four-circle X-ray diffractometer (Panalytical MRD X’Pert 3) with Cu *K*α radiation. The synchrotron based XRD measurements were performed at the beamline 1W1A of the Beijing Synchrotron Radiation Facility (BSRF). The wavelength of synchrotron X-ray is 1.24 Å. The microstructures of a Fe_3_N/VN bilayer were examined using JEM ARM 200CF electron microscopy at the IOP-CAS. The samples were prepared using Ga^+^ ion milling after mechanical thinning. Both HAADF and ABF imaging were performed in the scanning mode. Element-specific EELS mappings and dual-type EDX spectroscopy mappings were performed at the O *K*-, N *K*-, V *L*-, Fe *L*-edges from the regions of interest. XAS measurements were performed on a Fe_3_N/VN bilayer, a Fe_3_N single layer, a VN single layer, and a Fe/V bilayer using the total-electron yield (TEY) method. All structural characterizations were performed at room-temperature.

### Transport and magnetic characterizations

Transport properties were measured using standard van der Pauw geometry. The temperature and magnetic field dependent resistivity measurements were conducted using a 9T–PPMS. The *ac* current was kept at a minimum requirement of 1 μA to avoid Joule heating. The magnetic properties of a Fe_3_N single film and a Fe_3_N/VN bilayer were characterized by a SQUID equipped with the high-temperature unit. Both in-plane and out-of-plane magnetization were obtained at the variable temperatures and fields. For the diamagnetic measurements of a Fe_3_N/VN bilayer, the sample was zero-field cooled and measured during the sample warming up process at a field of 200 Oe.

### PNR measurements

PNR measurements were carried out at MR beamline of Chinese Spallation Neutron Source (CSNS), Dongguan, Guangdong Province. The size of a Fe_3_N/VN bilayer is 10 × 10 × 0.5 mm^3^. We performed PNR measurements at fixed temperatures of 3.5 and 15 K under an in-plane magnetic field of 1 T. These temperatures were chosen because the VN films stay in the superconducting (3.5 K) and normal state (15 K) during the PNR measurements. Additionally, we conducted a control measurement by fixing the temperature at 6.5 K. Similarly, the VN films can be switched between superconducting and normal state under magnetic fields of 2 kOe and 1 T, respectively. The specular neutron reflectivity (*R*) of a Fe_3_N/VN bilayer were recorded as a function of the wave vector transfer *q* (=4πsinα/λ), where α is the incident angle of neutron beam and λ is the wavelength of neutrons. Both *R*^+^ and *R*^–^ were recorded when neutrons with spins parallel or antiparallel to the applied fields (corresponding to the spin-up and spin-down neutrons), respectively. We fitted PNR data to a model (including the layer thickness and chemical roughness) that was obtained by XRR fitting using Parratt formalism. In these cases, the nSLD for the Fe_3_N and VN were fixed to their bulk values of ∼8.9 × 10^−6^ Å^−2^ and ∼5 × 10^−6^ Å^−2^, respectively.

### First-principles calculations

The first-principles calculations within the framework of DFT were based on Vienna *ab initio* Simulations Package (VASP) [55]. Projected augmented wave (PAW) and generalized gradient approximation (GGA) methods are adopted to treat the valence electrons-ion and exchange-correction effects, respectively [56]. To correctly describe the 3*d* electrons, the GGA + U method is employed with an effect Hubbard U value U_Fe_ = 1.5 eV for the Fe element and U_V_ = 3 eV for the V element [57]. Otherwise, a cutoff energy of 500 eV for plane-wave expansion and 6 × 6 × 1 *k*-point meshes are set for determining the most stable structure. In the meantime, the Fe_3_N/VN heterointerface is relaxed until the Hellmann-Feynman force acting on each atom becomes smaller than 1 × 10^−3^ eV/Å. Finally, the Heisenberg exchange coupling constant (*J*) between interfacial Fe and V elements is solved via comparing the energy between FM and AFM configurations (as shown when a 1×√3 × 1 supercell is constructed, Fig. S14).

## Supplementary Material

nwae107_Supplemental_File
